# OCT3-4 and SOX2 in Oral Lichen Planus Compared to Oral Leukoplakia: An Immunohistochemical Study

**DOI:** 10.7759/cureus.84837

**Published:** 2025-05-26

**Authors:** Vasileios Zisis, Nikolaos N Giannakopoulos, Athanasios Poulopoulos, Marc Schmitter, Dimitrios Andreadis

**Affiliations:** 1 Prosthodontics, Julius-Maximilians-Universität Würzburg, Würzburg, DEU; 2 Oral Medicine/Pathology, Aristotle University of Thessaloniki, Thessaloniki, GRC

**Keywords:** cancer biomarkers, cancer stem cells, head and neck squamous cell cancer, oct-4, oral lichen planus, sox 2, tumor stem cells

## Abstract

Background and objective

Cancer stem cells (CSCs) initiate carcinogenesis. This study aimed to examine them via immunohistochemistry in oral potentially malignant disorders (OPMDs).

Methods

The study involved 54 samples of OPMDs, which were compared with five cases of normal oral epithelium. The study was approved by the Ethics Committee of the School of Dentistry, Aristotle University of Thessaloniki, Greece (protocol number: 8/03.07.2019). The CSC-related proteins octamer-binding transcription factor (OCT)3-4 and SRY-related HMG-box (SOX)2 were examined with immunohistochemical methods. In the case of SOX2 and OCT3-4, the samples were evaluated through a scale of 1 to 3 depending on the presence of positive cells, and this scale was further multiplied by the intensity of staining (multipliers 1 and 2). The statistical analysis was performed with Fisher’s exact test, and the significance level was set at *p*≤0.05.

Results

The nuclear staining of OCT3-4 was not expressed in any of the samples. The nuclear staining of SOX2 was observed mostly in the basal and parabasal layer of the epithelium. Statistically significantly higher expression of SOX2 was observed in the erosive oral lichen planus (EOLP) group than in the reticular OLP (ROLP) group (*p*=0.05) and the mild and non-dysplastic leukoplakia group than in the reticular OLP group (*p*=0.024).

Conclusions

The characteristic expression of SOX2 in OLP suggests the presence of CSCs and might imply oral tumorigenesis even in lichenoid lesions. Erosive lichen planus outperformed the mild and non-dysplastic leukoplakia, regarding the expression of CSC biomarkers.

## Introduction

The octamer-binding transcription factor (OCT), specifically OCT4, plays a role in the development and progression of oral cancer [1]. Its expression levels are significantly correlated with chemoresistance and radiation resistance, which affects the treatment outcomes [2,3]. Silencing OCT4 may reduce tumor cell proliferation, migration, and invasion [1]. OCT4-knockdown shows promising results by increasing the sensitivity of oral squamous carcinoma cells to chemotherapy [2]. Oral potentially malignant disorders (OPMDs) are characterized by an imbalance in cellular transcription factors and pathways, involving OCT4, contributing to their malignant transformation [4]. Leukoplakia is linked with the expression of OCT4, which acts as a significant marker [5]. Targeting OCT4 offers a promising therapeutic goal as its dysregulation is often implicated in tumorigenesis across various types of cancer [6]. POU5F1B (POU class 5 homeobox 1B), a pseudogene associated with OCT4, has been identified as having oncogenic potential, particularly in cervical cancer [6]. The modulation of OCT4 expression through the silencing of POU5F1B has shown promising results in inhibiting cancer cell proliferation and migration [6]. Consequently, integrating OCT4-focused strategies into cancer treatment regimens could enhance the clinical outcome [6].

SOX (SRY-related HMG-box) family proteins also play a role in the progression of oral cancer. SOX8 is over-expressed in tongue squamous cell carcinoma (TSCC), where it binds to the promoter region of FZD7 (frizzled class receptor 7), hyper-regulating its expression. This activates the Wnt-signaling cascade, which maintains the stem cell-like state of cancer cells and the epithelial-mesenchymal transition process [7]. In cisplatin-resistant TSCC cells, the elevated levels of FZD7 correlate with the expression of markers such as N-cadherin, vimentin, and SOX2, while E-cadherin levels are reduced, contributing to the stem cell-like state of cancer cells and the epithelial-mesenchymal transition process, as well [7].

SOX9 is upregulated in TSCC cells, activating the Wnt/beta-catenin signaling cascade. This supports the self-renewal of cancer stem cells (CSCs) and contributes to the chemoresistance (such as to cisplatin) [7]. The interaction between SOX and OCT4 may determine the progression and therapeutic response of cancer. SOX2 is transcriptionally regulated by an enhancer containing a composite SOX-OCT element [6]. This SOX2-OCT4 complex upregulates genes that maintain pluripotency [8]. The presence of SOX2 is linked to the activation of signaling pathways such as the EGFR-Src-AKT [6]. RNA interference technologies like small interfering RNA (siRNA) and small hairpin RNA (shRNA) present a viable method to suppress SOX gene expression, reducing their oncogenic potential by targeting the mRNA of these genes [9]. The development of small-molecule inhibitors targeting SOX18 showed promise in disrupting tumor proliferation by inhibiting its DNA binding and transcription [9].

Both OCT4 and SOX2, as embryonic stem cell (ESC) markers, reprogram somatic cells into ESC-like states [10,11] and are master regulators for self-renewal and maintenance of the stem cell population in the undifferentiated state [12,13]. The primary aim of this study is to assess the potentially malignant character of oral lichen planus (OLP) by identifying the CSC biomarkers OCT4 and SOX2 and comparing their pattern of expression to oral leukoplakia (OL) and normal oral mucosa (NOM). OL constitutes the most prevalent OPMD, and any association of OLP with OL may support OLP’s malignant potential as well.

## Materials and methods

A total of 24 OLP samples, 30 OL samples, and five NOM samples were used in our experiment. We obtained the approval to conduct this study from the Ethics Committee of the School of Dentistry (protocol No. 8/03.07.2019). The samples were taken from the Department of Oral Medicine/Pathology, School of Dentistry, Aristotle University of Thessaloniki, Greece, during the period 2009-2019. The main inclusion criterion was the presence of an adequate quantity of paraffin-embedded tissue [14]. Furthermore, both male and female patients were included, with no restriction regarding the localization of the oral lesion.

**Figure 1:**
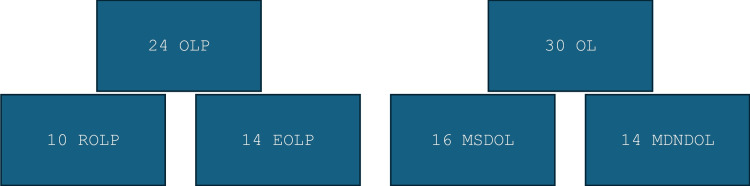
Categories and subcategories of our samples OLP: oral lichen planus; EOLP: erosive oral lichen planus; ROLP: reticular oral lichen planus; OL: oral leukoplakia; MSDOL: moderately and severely dysplastic OL; MDNDOL: mildly and non-dysplastic OL

The tissue samples were divided into the OLP group, the OL group, and the NOM group. The OLP group was subdivided into the reticular OLP group (ROLP) and the erosive OLP group (EOLP). The OL group was subdivided into the moderately or severely dysplastic OL group (MSDOL) and the mildly or non-dysplastic OL group (MDNDOL) according to the WHO 2005 binary classification system for OL [15]. In total, 10 cases of ROLP, 14 cases of EOLP, 16 cases of MSDOL, 14 cases of MDNDOL, and five cases of NOM were included (Figure 1).

The immunohistochemical method required the CSCs’ protein-biomarkers anti-OCT3-4 (Santa Cruz Biotechnology, Dallas, TX) and anti-SOX2 (Santa Cruz Biotechnology), as well as the Dako Envision Flex+ system (Dako Denmark A/S, Glostrup, Denmark).

The evaluation of the nuclear staining of SOX2 and OCT3-4 was obtained as a histochemical score by calculating the percentage of positive cells, and then classifying this percentage into a scale of 0-3 and then multiplying it by 1 (weak staining) or 2 (strong staining) depending on the intensity of the staining. The staining was deemed to be successful when the cytoplasm, membrane or nucleus was colored brown [16].

Statistical analysis was performed using the SPSS Statistics software 2017 (IBM Corp., Armonk, NY) and involved Fisher’s exact test. The significance level was set at a p-value ≤0.05.

## Results

**Figure 2:**
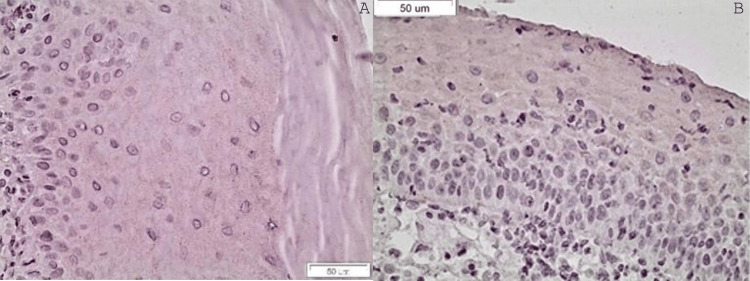
(A) Absent staining of OCT3-4 in a case of ROLP (x40). In case of positive staining, nuclear staining was to be expected. (B) Absent staining of OCT3-4 in a case of EOLP (x40). In case of positive staining, nuclear staining was to be expected ROLP: reticular oral lichen planus; EOLP: erosive oral lichen planus

Regarding the staining of OCT3-4, all the samples were scored as 0. OCT3-4 was not expressed at all in any of our samples (Figures 2A-2B). Therefore, further statistical analysis was not required.

**Figure 3:**
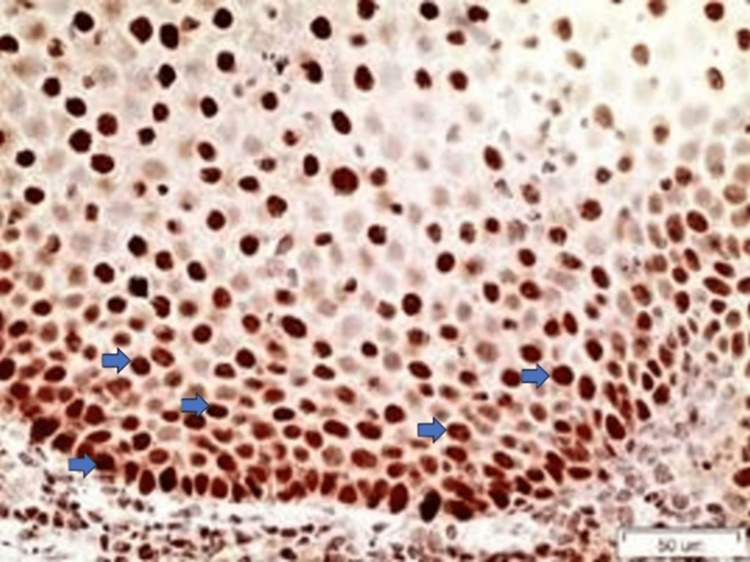
SOX2 staining in a case of ROLP (x40). The lower third of the epithelium is stained and positively stained cells are noticed (blue arrows). Nuclear staining is observed ROLP: reticular oral lichen planus

Regarding the staining of SOX2, all of the ROLP samples were scored as 1 (Figure 3).

**Figure 4:**
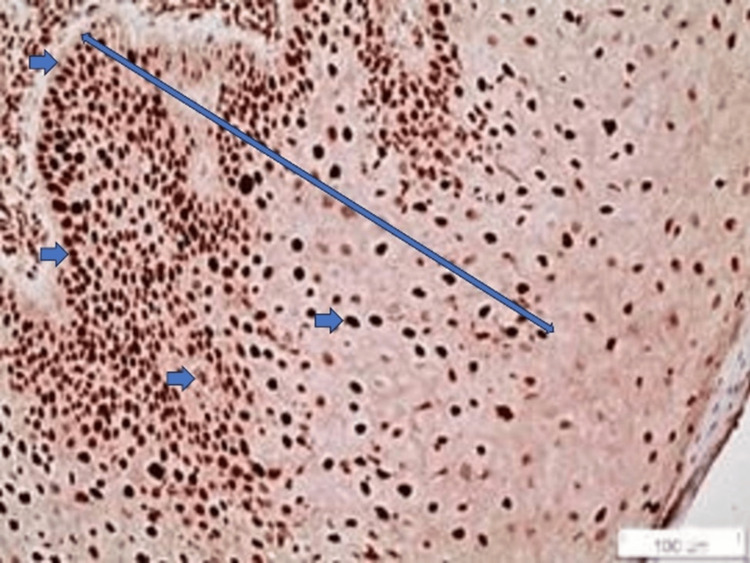
SOX2 staining in a case of EOLP (x20). The staining of the lower and middle third of the epithelium is noticed (double-edged arrow). In addition, positively stained cells are noticed (blue arrows). Nuclear staining is observed EOLP: erosive oral lichen planus

Eight samples of the EOLP were scored as 1, five samples of the EOLP were scored as 2, and one sample of EOLP was scored as 4 (Figure 4).

Ten samples of the MSDOL were scored as 1, three samples of the MSDOL were scored as 2, and three samples of the MSDOL were scored as 4. Eight samples of the MDNDOL were scored as 1, and six samples of the MDNDOL were scored as 2. All the samples of the normal oral epithelium were scored as 1.

Based on the histochemical scores and the statistical analysis, the following observations emerge (Table 1): (A)OCT3-4 was not expressed in any of the samples. Therefore, no statistical test was performed. (B) There was a statistically significantly higher expression of SOX2 in the EOLP than in the ROLP (Fisher’s exact test, *p*=0.05). There was a statistically significantly higher expression of SOX2 in the MDNDOL than in the ROLP (Fisher’s exact test *p*=0.024).

**Table 1:** Summary of the statistically significant results in our study ROLP: reticular oral lichen planus; EOLP: erosive oral lichen planus; MDNDOL: mildly and non-dysplastic oral leukoplakia

SOX2	EOLP	MDNDOL
ROLP	*P*=0.05	*P*=0.024

## Discussion

The term "oral potentially malignant disorder (OPMD)" refers to oral mucosal lesions at risk for a higher probability of transforming into malignancy compared to healthy mucosa [17]. It is preferred over the older terms of precancerous and premalignant lesions in the literature. Almost all oral cancers derive from precancerous lesions [17]. The most common form of OPMD is OL [18]. OLP constitutes a rarer form of OPMD [19]. CSCs appear in tumor initiation, metastasis, drug resistance, and relapse, thereby controlling the transition from OPMD to cancer [20]. As to what extent OLP is premalignant [21], and which clinical subtypes in particular, are still being investigated, and the identification of CSCs' biomarkers is warranted for that purpose.

CSCs drive the progression from potentially malignant lesions to oral squamous cell carcinoma (OSCC); hence, their presence also indicates that a condition is potentially malignant. Evidence concerning biomarkers OCT3-4 and SOX2 available so far is in the following lines: OCT4 is bound up in oncogenic processes [12], metastasis [22], and epithelial to mesenchymal transition [23]. The expression of OCT4 in OSCC is much higher in the surrounding tissues compared with both normal tissues and the tumor itself, and high levels of OCT4 expression in OSCC are characteristic of an early stage of disease and thus a more benign clinical course [24]. The presence of OCT3-4 plays a key role in the development of delayed neck metastasis through increased cell motility and invasiveness [25]. The expression of SOX2 in OSCC was found to be higher both in the tumor and the peritumoral vessels [26]. SOX2-positive cells are more in OSCC and oropharyngeal SCC [27]. Overexpression of SOX2 enhances invasion, simultaneously silencing drug resistance and anti-apoptotic genes [28].

In our study, we found that the moderately or severely dysplastic leukoplakia expressed SOX2 to a similar extent to EOLP. A logical assumption to be drawn is that the two entities do not behave differently on a molecular basis. Islam et al. noticed concurrent expression of SOX2 and OCT4 in the immunofluorescence assay of lichenoid lesions and leukoplakia, supporting their precancerous nature [29]. These findings, regarding SOX2, are similar to ours on a different level of analysis (immunofluorescence vs. immunohistochemistry) but contradict our reported absent staining of OCT3-4 in lichenoid lesions and leukoplakia on the immunohistochemical level. The mildly dysplastic or non-dysplastic leukoplakia group expressed OCT3-4 similarly to the reticular lichen planus group, while expressing SOX2 more than the reticular lichen planus group. A logical assumption to be drawn is that the two entities behave partially differently on a molecular basis. Finally, erosive lichen planus expressed more SOX2 than reticular lichen planus.

These findings could be interpreted as follows: on a clinical level, it appears that patients with erosive lichenoid lesions should undergo stricter follow-ups, just like patients diagnosed with OL with moderate and or severe dysplasia. On an experimental level, these findings require further support through experimentation in more samples and ideally by comparing different erosive lichenoid lesions among themselves (due to materials, drugs, in terms of Greenspan syndrome, in terms of Systemic erythematosus lupus, etc.).

Limitations

This study has a few limitations. They include the lack of sample size calculation, the lack of HPV status of the patients involved, as well as the lack of follow-ups to observe which cases evolved into higher degrees of dysplasia.

## Conclusions

OLP belongs to OPMDs, and the expression of CSC biomarkers allows for a better evaluation of the lesions, regarding their prognosis and potentially malignant potential. Erosive lichen planus outperformed the mildly and non-dysplastic leukoplakias, regarding the expression of CSC biomarkers. This finding should be taken into account when deciding the frequency of follow-ups for the patients. Moderate dysplasia, severe dysplasia, and erosive lichenoid lesions should be monitored more closely than their mild and reticular counterparts.
